# Posterior ankle impingement syndrome and os trigonum relationship in children

**DOI:** 10.14744/nci.2021.22587

**Published:** 2022-02-08

**Authors:** Merter Keceli

**Affiliations:** Department of Pediatric Radiology, Health Sciences University, Konya City Hospital, Konya, Turkey

**Keywords:** Children, os trigonum, posterior ankle impingement, radiology

## Abstract

**Objective:**

The os trigonum is a common cause of posterior ankle impingement in children and adults. Its incidence in the medical literature is controversial. The aim of the study is to determine the incidence of os trigonum, medullary imaging features and size in pediatric patients with suspected posterior ankle impingement.

**Methods:**

Fifty-five children (4–16-years-old; 16 girls,) who underwent magnetic resonance ımaging (MRI) with a pre-diagnosis of posterior ankle impingement syndrome (PAIS) were included in the study. Their ankle MRI and lateral radiograms were retrospectively reviewed. The relationship between os trigonum type, size, medullary signal characteristics, and PAIS development was investigated. Skeletal maturity was graded based on the ossification pattern of the calcaneal apophysis. The possible relationship between skeletal maturity and PAIS caused by os trigonum was investigated.

**Results:**

Among the 55 participants, there were 14 participants diagnosed with PAIS (9 boys, mean age 15±1.2 years). There was no connection between the os trigonum type, its medullary signal, gender, and PAIS clinical picture (p>0.05). The os trigonum size causing PAIS was 9±3.4 mm on average. There was a statistically significant relationship between PAIS complaints and os trigonum size (p=0.04). There was no significant relationship between calcaneal stage and PAIS clinical picture (p=0.669). When the os trigonum was fused, all participants were at calcaneal stage 3 or higher.

**Conclusion:**

MRI is superior in detecting fusion of the os trigonum with the talus, the medullary signal of ossicle, and PAIS findings. The most important factor in the development of PAIS is the size of the os trigonum.

**P**osterior ankle impingement syndrome (PAIS) is a range of clinical disorders characterized by posterior ankle pain during plantar flexion [1]. PAIS has been more widely recognized among athletes. PAIS, may be due to both bone and soft tissue lesions and anatomical variants [2]. Osseous lesions include the Stieda process, os trigonum, osteophytes, osteochondral lesion, loose bodies, chondromatosis, and subtalar coalition [3, 4]. The majority of the posterior impingement syndromes are related to the posterior talus. The secondary ossification center in the posterolateral part of the talus occurs between the ages of 8–15 and merges with the talus within 1 year [5]. Os trigonum may not fuse with the talus in 7% of cases [5, 6]. In addition, this ossification center may remain prominent with the so-called “Stieda’s process.” Clinically, the PAIS presents with chronic pain and swelling within the posterior ankle. This syndrome occurs in activities that cause excessive plantar flexion, such as ballet, football, soccer, and downhill running [5]. There are two main predicted mechanisms in the development of posterior impingement: acute plantar hyperflexion injury and chronic recurrent microtrauma [7, 8]. The similarity between the two mechanisms revolves around the posterior soft tissues, which may become secondarily hypertrophied and compressed between the posterior talus and the calcaneus. The presence of an osseous body can narrow this anatomic space, which has led to its other name of os trigonum syndrome [5]. This increased compression leads to damage to the regional tendons and ligaments. The most commonly involved flexor hallucis longus tendon [8]. Important differentials include Achilles tendinosis/tendon tear, arthrosis, acute posterior talar process fractures, flexor hallucis longus tenosynovitis, Haglund’s syndrome, osteochondral lesions, and retrocalcaneal bursitis [9]. Assessment by conventional radiogram may be normal, but lateral radiogram should be carefully examined to assess the presence of a Stieda process or an os trigonum. Magnetic resonance imaging (MRI) with its superior soft-tissue contrast feature plays an important role in the evaluation of posterior ankle pain. Bone marrow edema in the talus, calcaneus or os trigonum is the best guide [5, 6]. Other features include increased signal at the synchondrosis, associated synovitis, and thickening of the posterior ligaments, as well as the possibility of a posterior subtalar or tibiotalar ganglia being present.

PAIS is manifested by posterior ankle pain that develops during plantar flexion. Repetitive plantar flexion, overuse, recurrent trauma, congenital anatomic variations lead to this syndrome [10]. Congenital variations include os trigonum, Stieda process, tubercle of posterior talar process, and the prominent posterior downward slope of the tibia [11].

Soft tissue lesions are flexor hallux longus tenosynovitis, synovitis, joint capsule, and compression of abnormal muscles [4]. Accessory ossicles are common variations in all age groups. The ossicles around the feet originate from secondary ossification centers that are not fused. Accessory ossicles are generally asymptomatic in all age groups and are recognized incidentally in radiological examinations [11, 12]. They lead to symptoms according to their location. Accessory ossicle located in the posterior aspect of the ankle, os trigonum, is the most common of these formations. These ossicles originating from the posterior part of the talocalcaneal joint causes edema with repeated trauma in the paratendinous area adjacent to the talocalcaneal joint. In the medical literature, the incidence of os trigonum in symptomatic cases reported at rates ranging from 2% to 26% [11–13]. It is necessary to differentiate the Stieda process from the os trigonum, which is formed by the fusion of the secondary ossification center with the rest of the talus in the posterolateral direction of the talus [14].

**Highlight key points**

• Posterior ankle impingement syndrome (PAIS) is a common cause of ankle dysfunctions due to physical activity in childhood and adolescence.
• Os trigonum is a common variation leading to posterior ankle impingement syndrome. While types of Os trigonum do not make a significant difference for PAIS formation, ossicular size is an important factor.
• MRI is the most useful imaging method in revealing PAIS and its causes.

The aim of this cohort study is to determine the incidence, medullary imaging features, and size of os trigonum in pediatric patients with suspected posterior ankle impingement.

## Materials and Methods

Before starting to collect data for the study, local ethics committee approval (KTO Karatay University Faculty of Medicine Local Ethics Committee; 03.11.2020/006) was obtained. Unilateral 360 ankle MRI examinations performed on children aged 4–16 years who were referred to the imaging department between January 2015 and June 2020 due to foot-ankle pain, trauma, and infection pathologies were re-evaluated. Children with a history of hip, knee, foot-ankle trauma or surgery were excluded from the study. Children with metabolic and endocrine system diseases were not included in the study. Thus, the number of participants examined for foot-ankle pain fell to 112. It was seen that the number of children whose etiology of posterior ankle pain was investigated from hospital information system was 75. In other 37 pediatric patients, it was observed that intra-articular foreign body, bone-bone marrow involvement due to hematological and oncological malignancies, synovial pathology, and the etiology of dorsal foot pain were investigated. Fifty-five of 75 participants who had lateral ankle-ankle radiography in 1 month in addition to MRI were included in the study. Clinical findings, treatment practices, and results of these participants were completely recorded by the clinicians in the hospital information system. Demographic characteristics of all participants were similar. There were no professional athletes and heavy laborers among the participants.

MRI images were acquired using a 1.5-T unit (Siemens Magnetom Area, Siemens Healthineers AG, Erlangen, Germany) and standard ankle coil. Fast spin-echo T1-weighted and T2-weighted images were obtained in the routine axial plane, oblique axial-coronal plane, and oblique axial-sagittal plane. MRI parameters were listed as follows: repetition time/echo time, 400/12 ms for T1-weighted and 3600/85 ms for T2-weighted images; echo train length, 3 for T1-weighted and 20 for T2-weighted images; field of view 180 mm; slice thickness 3 mm; and interslice gap 1,5 mm. Those with protocol errors and artifacts in MRI examinations were excluded from the study.

Bone age evaluations of the participants with os trigonum were performed using calcaneal epiphysis staging from lateral radiographs of the ankle and foot [15].

All radiological reassessment was performed by a pediatric radiologist with 10 years of professional experience. MRI images were reassessed, blinded for patient characteristics for the talocalcaneal joint, the presence of medullary edema in the posterior part of the talus and calcaneus, the presence of osteophytic formation originating from the talus or calcaneus, the presence of os trigonum variation, and the presence of fluid in the paratendinous area in front of the Achilles Tendon were re-evaluated by same radiologist. These changes identified in imaging were used for the diagnosis of PAIS [10, 11, 16]. The participants with os trigonum were examined in terms of ossicle size, presence of medullary sclerosis or edema in the ossicle, and presence of PAIS findings from hospital information system records. The ossicle’s size, along with other parameters, was measured electronically on MRI by the same radiologist.

**Figure 1.**
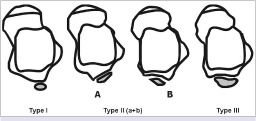
The drawing shows the relationship between os trigonum and talus: Type I: While the talar tubercle is in its normal appearance, with a separate ossicle, Type II: The ossicle located medially or laterally (A or B), as part of the talar tubercle, Type III: The ossicle developed in this area without the development of the talar tubercle.

The os trigonum was grouped into three different types in X ray, based on Zwiers et al.’s [7] classification; Type I: While the talar tubercle is in its normal appearance, with a separate ossicle, Type II: The ossicle as part of the talar tubercle, Type III: The ossicle developed in this area without the development of the talar tubercle (Fig. 1). The detected os trigonums were grouped according to this method.

The relationship between os trigonum and the presence of PAIS was evaluated. The possible connection between the calcaneal epiphyseal stage and os trigonum size, this osscile’s MRI signal features were investigated.

### Statistical Analysis

In calculating the sample size of this study, the power (power of the test) for each variable was determined as at least 80% and the 1^st^ type error was 5%. Whether the continuous measurements in the study were normally distributed or not was checked with Kolmogorov-Smirnov (n>50) and Skewness-Kurtosis tests, and parametric tests were applied because the measurements were normally distributed. Descriptive statistics for continuous variables in our study; mean, standard deviation, minimum, maximum; For categorical variables, it was expressed as numbers and percentages. Independent T-test and One Way Analysis of Variance were used to compare all continuous measurements according to groups. Pearson correlation coefficients were calculated to determine the relationships between measurements. Chi-square test was used to determine the relationship between categorical variables. In the calculations, the statistical significance level was taken as (a) 5% and the SPSS (IBM SPSS for Windows, ver. 24, Chicago, IL, USA) statistics package program was used for calculations.

### Results

The mean age of 55 participants (16 girls; 39 boys) whose unilateral ankle images were selected was 13.7±0.23 years. Average age was 14±2.2 years for girls and 13.6±2.7 years for boys (Table 1).

**Table 1.** Comparison of age, os trigonum size and calcaneal stage by gender

	N	Mean	SD	Min	Max	*p
Age (year)						0.451
M	39	13.54	2.371	8	16
F	16	14.06	2.205	9	16	
Total	55	13.69	2.316	8	16	
Size (mm)						0.942
M	39	7.31	2.647	3	14
F	16	7.25	2.745	3	15	
Total	55	7.29	2.650	3	15	
Calcaneal stage						0.662
M	39	3.85	0.779	2	5
F	16	3.94	0.443	3	5	
Total	55	3.87	0.695	2	5	

*: Significance levels according to independent T-test results; M: Male; F: Female; SD: Standard deviation; Min: Minimum; Max: Maximum.

When gender was ignored, 14 of the participants had type I (25%), 30 of them had type II (55%) (12 of 30 children type IIa, 18 of 30 children type IIb), 11 of them had type III (20%) os trigonum. Of the 14 participants who were diagnosed with radiological PAIS, 11 had type II (78%) and 3 had type I (22%) os trigonum. There was no statistical relationship between ossicle type and gender (p=0.554). In addition, a statistically significant relationship was not observed between the development of PAIS and the os trigonum type (p=0.90).

The mean age of 14 participants diagnosed with PAIS among 55 participants was 15±1.2 years. Of the 14 cases, nine were boys (64%), five were girls (36%). There was a significant statistical relationship between PAIS and os trigonum size (p=0.04). The average size according to the os trigonum measurements made in the anteroposterior direction in all participants was 7.3±2.6 mm. This size was 9±3.4 mm and significantly larger in participants with PAIS (Table 2).

**Table 2.** Comparison of age, os trigonum size and calcaneal stage by talus-os trigonum in terms of PAIS

	N	Mean	SD	Min	Max	*p
Age (year)						0.005
PAIS–	41	13.20	2.400	8	16
PAIS+	14	15.14	1.231	12	16	
Total	55	13.69	2.316	8	16	
Size (mm)						0.004
PAIS–	41	6.71	2.065	3	11
PAIS+	14	9.00	3.442	3	15	
Total	55	7.29	2.650	3	15	
Calcaneal stage						0.219
PAIS–	41	3.80	0.715	2	5
PAIS+	14	4.07	0.616	3	5	
Total	55	3.87	0.695	2	5	

*: Significance levels according to independent T-test results; PAIS: Posterior ankle impingement syndrome; SD: Standard deviation; Min: Minimum; Max: Maximum.

The edema and sclerosis signal was sought by evaluating the medullary area signal in the os trigonum. When gender difference was ignored, medullary sclerosis in the ossicle of 28 participants (50.9%) and medullary edema in the ossicle of 12 participants (21.8%) was detected. Of the os trigonums detected in participants with radiological and clinical diagnosis of PAIS, 5 (36%) had medullary sclerosis and 4 (28%) had partial medullary edema. There was no change in the medullary signal in the five ossicles (36%). There was no statistical relationship between participant’s age, gender, calcaneal stage, and presence of os trigonum edema and sclerosis on MRI (respectively p=0.281–0.829).

The number of participants whose os trigonum fusion was completed was 22 (40%). Of these participants, 12 were boys (55%) and ten were girls (45%). The mean age of completed os trigonum fusion was 15±1.8 years. The average age was found to be 15±0.8 years for girls and 15±1.4 years for boys. The fused os trigonum was type I in 4 (18%), type II in 14 (64%), and type III in 4 (18%) of the participants. When gender was ignored, the calcaneal ossification stage determined was on average 3.8±0.6. There was no difference in the grading of calcaneal skeletal maturity stages during fusion between boys (mean stage 3.8±0.8) and girls (mean stage 3.94±0.4) (p=0.669). When os trigonum fusion was detected, all participants were at stage 3 or higher (Table 3).

**Table 3.** Comparison of age, os trigonum size and calcaneal stage by talus-os trigonum in terms of PAIS

	N	Mean	SD	Min	Max	*p
Age						<0.001
Fusion–	33	12.70	2.325	8	16
Fusion+	22	15.18	1.296	12	16	
Total	55	13.69	2.316	8	16	
Size						0.001
Fusion–	33	6.33	2.160	3	11
Fusion+	22	8.73	2.711	5	15	
Total	55	7.29	2.650	3	15	
Calcaneal Stage						<0.001
Fusion–	33	3.58	0.614	2	5
Fusion+	22	4.32	0.568	3	5	
Total	55	3.87	0.695	2	5

*: Significance levels according to independent T-test results; PAIS: Posterior ankle impingement syndrome; SD: Standard deviation; Min: Minimum; Max: Maximum.

As the calcaneal stage increased, a higher rate of fusion was seen (p<0.001). When the relationship between os trigonum fusion and its size was evaluated, the mean size of the fused ossicles was found to be 8.7±2.7 mm. The non-fused ossicle size was on average 6.3±2.1 mm. A significant relationship was found between talar fusion and os trigonum size (p<0.001). It was seen that the possibility of talar fusion increases as the ossicle grows.

### Discussion

The os trigonum is known as one of the most common causes of PAIS. However, there is no consensus on the definition and incidence of the os trigonum. In our study, we aimed to determine the incidence, medullary imaging characteristics, and size of os trigonum in pediatric patients with suspected posterior ankle impingement. According to our results, os trigonum type, medullary signal of the ossicles, gender were not associated with PAIS development. We found a significant correlation between PAIS complaints and os trigonum size. There was no relationship between the calcaneal stage and PAIS clinical picture.

**Figure 2.**
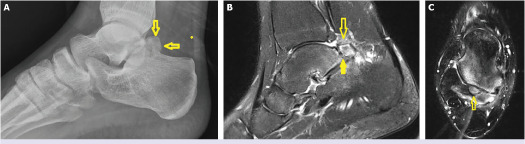
Fifteen-year-old boy with posterior ankle impingement syndrome. **(A)** On the lateral of the foot radiogram, the os trigonum with a sclerotic signal close to 1 cm is seen in the posterior neighborhood of the talus (arrow). **(B, C)** There is no connection between the os trigonum and the talus in the fat saturated T2W sagittal and axial plane images. The ossicle, which is the signal of medullary edema on MRI, is seen as a separate bone structure (arrows). In addition to os trigonum edema, talus and soft edema can be selected on MRI

**Figure 3.**
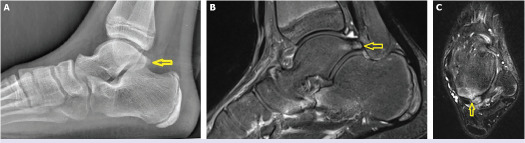
Nine-year-old boy with posterior ankle pain. **(A)** On lateral of the foot-ankle radiogram, the os trigonum is found to be fused with the talus (arrow). **(B, C)** Sagittal and axial fat-saturated T2W images can be seen separately from the os trigonum talus with clear boundaries (arrows). In the axial plan, it can be seen that the os trigonum joins the talus with a small bone connection (arrow in C).

In the study of Zwiers et al. [7], the prevalence of os trigonum was reported between 1.7% and 12.7%. In their study, they found the incidence of os trigonum as 30.3% in the patient population with a diagnosis of PAIS and 23.7% in those without a diagnosis of PAIS. In this current study, 25% of the participants with os trigonum had PAIS findings. Consistent with the results of previous studies, no association was found between the presence of os trigonum and gender (p>0.554). Knapik et al. [17], in their study by examining radiograms, stated the mean age of fusion as 17. In our study, the mean complete fusion of the os trigonum in men was 15±1.4; it was observed that the girls were around 15±0.8-years-old. In this study, the presence and absence of the cartilage connection between the ossicle and the talar tubercle were determined precisely because the fusion development was evaluated by MRI (Fig. 2, 3). Therefore, the connection of the medullary areas of both bone structures could clearly visible. Knapik et al. [17] report that they think that the os trigonum is an extension with a cartilage connection between it and the talus, rather than a separate bone structure. However, in current study, participants were determined that these ossicles identified in MRI images are bone structures separate from the main bone structure, and there is no connection between talus tubercles and ossicles. Based on this finding, it was thought that the os trigonum was a different bone formation.

There was no difference in the grading of skeletal maturity according to calcaneal ossification stages during fusion between boys and girls (p=0.5). All participants were at stage 3 or higher during os trigonum fusion (Table 3). The reason why this last finding is different from the previous study is the evaluation of fusion by radiogram in this study [17]; in our study, the use of MRI could be.

When os trigonum is symptomatic, primary care includes: conservative management with reducing or changing the activity, supplementary insoles, safety shoes, nonsteroidal anti-inflammatory drugs. According to the results of the studies of Knapik et al. [17]; conservative treatment is recommended in symptomatic adolescents, as ossicular fusion is seen in half of these cases within 12 months. They reported that conservative treatment should be chosen also in cases with lower bone maturity. Surgical recommended only when there is pain and swelling it continues despite non-surgical treatment [18].

This cohort study is superior in that evaluation of the ossicular presence, size, medullary signal, talar fusion, and effects on surrounding tissues is done by examination on MRI images. Another advantage is that the diagnosis of PAIS can be definitively identified radiologically. This study does have a number of limitations. The number of participants who could be included in the study was low due to the small number of ankle MRI examinations performed for PAIS. Another limitation is that the side of the ankles examined could not be standardized. During reassessment, the ankles from which the images were taken were either the right or the left side. An important limitation was that all imaging was obtained from the symptomatic side and the evaluations were unilateral. When evaluating the os trigonum medullary signal change, it was not known how long after the onset of PAIS in most of the participants imaging was performed. Since there is no statistical relationship between medullar signal change and other parameters, this has a limited effect on the result.

### Conclusion

Os trigonum is among the causes of PAIS in all age groups. Os trigonum should be investigated when evaluating posterior ankle pain in children and adolescents. MRI examination is superior in detecting fusion of the os trigonum with the talus, the medullary signal of ossicular formation, and PAIS findings. While types of Os trigonum do not make a significant difference for PAIS clinic, ossicular size is an important factor. As its size increases, the fusion of the ossicle and talus increases. According to the results of the current study, it is seen that fusion develops as the age increases. It can be said that PAIS, which develops due to the os trigonum, develops more frequently in older children with larger and fused ossicles.
